# Using synchrotron based ATR-FTIR, EXAFS, and XRF to characterize the chemical compositions of TSP in industrial estate area

**DOI:** 10.1016/j.heliyon.2024.e39215

**Published:** 2024-10-12

**Authors:** Siwatt Pongpiachan, Kanjana Thumanu, Waraporn Tanthanuch, Duangjai Srisamut, Jureerat Pradabsri, Muhammad Zaffar Hashmi, Yan Sun, Saran Poshyachinda

**Affiliations:** a National Astronomical Research Institute of Thailand (Public Organization), 260 Moo 4, T. Donkaew, A. Maerim, Chiang-Mai, 50180, Thailand; b Synchrotron Light Research Institute (Public Organisation), 111 Moo 6, University Avenue, Muang District, Nakhon Ratjasima, 30000, Thailand; c NIDA Center for Research & Development of Disaster Prevention & Management, School of Social and Environmental Development, National Institute of Development Administration (NIDA), 118 Moo 3, Sereethai Road, Klong-Chan, Bangkapi, Bangkok, 10240, Thailand; d Department of Chemistry, COMSATS University, Islamabad, 44000, Pakistan; e Key Laboratory of Behavioral Science, Institute of Psychology, Chinese Academy of Sciences, 4A Datun Road, Chaoyang District, Beijing, 100101, China

**Keywords:** Synchrotron radiation (SR), Total suspended particulate (TSP), FTIR (Fourier transform infrared) spectroscopy, X-ray fluorescence (XRF), Health risk assessment

## Abstract

In Thailand, the Map Ta Phut Industrial Estate (MTPIE), a prominent industrial hub, has substantial environmental and health issues caused by industrial pollution. This study uses advanced synchrotron-based techniques, such as Attenuated Total Reflectance Fourier Transform Infrared (ATR-FTIR), Extended X-ray Absorption Fine Structure (EXAFS), and X-ray Fluorescence (XRF), to fully examine the chemical make-up of total suspended particulate (TSP) in the given area. Notable findings include the detection of remarkably high enrichment factors for magnesium and sulfur, indicating the presence of industrial operations. Additionally, we found that magnetite, which accounts for an average of 40 % of the total iron oxides in the samples, is the main iron oxide. The study also highlights about how calcium carbonate and different organic functional groups are found in large amounts, which shows that industrial emissions and natural sources are connected in a complex way. The findings underscore the susceptibility of children to TSP exposure, revealing increased rates of inhalation and significant health hazards. In order to safeguard public health in industrial areas such as MTPIE, it is imperative to implement more sophisticated pollution control techniques and maintain ongoing environmental monitoring.

## Highlights

• Magnetite exhibited the largest contribution of iron oxides in TSP collected at industrial area.
• Both industrial activities and crustal emissions play a crucial role in governing the atmospheric contents of selected elements.
• The exceptionally high Log(*EF*) values of Mg and S can be ascribed to industrial activities.
• This study highlights the susceptibility of infants to TSP exposure due to their elevated levels of inhalation and ingestion.

## 1 Introduction

The 2009 Map Ta Phut Industrial Estate (MTPIE) air pollution case in Thailand was a pivotal moment in the country's environmental regulatory framework. The temporary suspension of operations of key industrial facilities in MTPIE by the Central Administrative Court indicates a change in focus towards giving greater importance to safeguarding public health and protecting the environment, rather than pursuing immediate economic benefits from industrial activity [1]. This order not only temporarily terminated industrial operations but also essentially postpone any future industrial growth in the region, establishing a standard for future environmental governance decisions in Thailand. The instance exemplified the idea of route dependence in Thai pollution control, emphasizing the long-lasting impact of the 1975 environmental law [2]. This law gave equal power to both the Ministry of Industry (MOI) and other environmental authorities.

The MOI implemented measures such as "Eco-industrial Towns" and mechanisms for regulating volatile organic compound emissions in response to the lawsuit, demonstrating the impact of institutional authority on pollution management tactics [3]. The MTPIE has emerged as a central hub for environmental movement in Thailand. Local communities and environmental non-governmental organizations have united to oppose industrial pollution, asking for more stringent environmental legislation and its effective implementation [4]. The Thai government has responded to this popular pressure by taking action. The initiatives encompass the enforcement of more stringent rules, the execution of environmental impact studies, and the augmentation of industrial emissions monitoring. Notwithstanding these endeavors, obstacles persist. The MTPIE and other industrial zones in Thailand face a continuous challenge in finding the most appropriate source apportionment technique under the context of complex potential sources of air pollutants.

In spite of numerous source apportionment studies associated with the air pollutants in Thailand, little is known about the chemical characteristics of aerosols in the MTPIE [[5], [6], [7], [8]]. Furthermore, there are limited studies on the application of synchrotron radiation for characterizing chemical compositions of aerosols in Thailand and other countries [[9], [10], [11], [12], [13], [14], [15]]. Conventional techniques used to examine air pollution in industrial regions, particularly the chemical makeup of Total Suspended Particulates (TSP), frequently fail to offer a complete understanding. Thankfully, researchers can benefit greatly from synchrotron-based techniques such as Synchrotron Radiation based Extended X-ray Absorption Fine Structure (SR-EXAFS), Synchrotron Radiation based Attenuated Total Reflectance-Fourier Transform Infrared (SR-ATR-FTIR), and Synchrotron Radiation based X-ray Fluorescence (SR-XRF) [9,10,[16], [17], [18], [19], [20], [21], [22]]. Each of these strategies possesses unique capabilities.

Synchrotron Radiation based X-ray Absorption Fine Structure (SR-XAFS) spectroscopy is a widely used method for studying the chemical forms of iron (Fe) in aerosols. Several studies have utilized this technique to investigate Fe speciation in aerosols [16,21,23]. The SR-XAFS examination of bulk samples provides direct insights into the average Fe speciation, which is anticipated to be associated with the proportion of soluble Fe. The SR-XAFS technique consists of two components: Synchrotron Radiation based X-ray Absorption Near-Edge Structure (SR-XANES) and SR-EXAFS [17,20]. Nevertheless, SR-EXAFS requires extremely accurate determination of the proportional alteration in X-ray absorption, requiring a precision superior to 10^−3^. Due to the low quantity of material captured on filters, the requirement typically makes SR-EXAFS unsuitable for aerosol samples, resulting in weak signals [18]. Therefore, numerous investigations have chosen SR-XANES spectroscopy instead of SR-EXAFS for determining the Fe speciation in aerosols since it is comparatively easier to measure. Although there are some disadvantages, SR-EXAFS offers precise and intricate insights about the exact chemical condition of elements present in aerosols [19]. This encompasses the oxidation state, the quantity of adjacent atoms, and the lengths of the bonds [22]. This data aids in the identification of the precise compounds that include these components and facilitates comprehension of their potential environmental consequences. In addition, SR-EXAFS enables researchers to selectively analyze specific elements in a sample, even when they are present in little amounts. On the other hand, SR-ATR-FTIR is highly effective in determining the specific functional groups found in organic molecules within the aerosols [10,24,25]. This aids in distinguishing between different types of organic functional groups (OFGs), such as hydrocarbons, alcohols, phenols, carboxylic acids, esters, amides, and amines [10]. Additionally, it provides reasonably quick analysis times, which enhances its efficiency in screening several samples. On the contrary, SR-XRF offers a quick and precise investigation of the elemental makeup of the TSP [9,26,27]. It has the ability to identify a diverse array of components, including those that are present in very small quantities. SR-XRF analysis is characterized by its non-destructive nature, which allows for subsequent investigation of the sample using additional techniques [28].

This integrated methodology provides a comprehensive analysis of the chemical makeup of TSP, encompassing both inorganic and organic constituents. This information is essential for identifying the potential origins of pollutants within the industrial estate and creating precise pollution management plans that target specific contaminants of concern. The synchrotron-based techniques are highly effective tools for enhancing environmental monitoring and pollution control tactics in industrial regions. Overall, the main principles of this study are (*i*) to utilize the synchrotron-based techniques (i.e. SR-EXAFS, SR-ATR-FTIR, and SR-XRF) for chemically characterizing TSP collected at MTPIE, (*ii*) to assess the temporal variation of both inorganic and organic components in TSP, and (*iii*) to employ multivariate statistical techniques for identifying potential sources of particulate air pollutants in the largest industrial estate of Thailand.

## 2 Materials & methods

### 2.1 Sampling site & TSP collection

The MTPIE in Thailand exemplifies the conflict between economic advancement and environmental preservation in developing countries [29]. The MTPIE, founded in 1989, has evolved into a central location for large-scale enterprises, drawing in petrochemical, oil refining, and manufacturing facilities [30]. Nevertheless, the swift expansion has resulted in negative consequences, as citizens and environmental organizations express apprehension regarding the contamination of air, water, and soil caused by industrial operations [4]. The emission of dangerous chemicals and particulate matter from factories is a major concern, as it is associated with respiratory issues among inhabitants [31]. Rivers and groundwater have been affected by industrial discharges, and soil contamination has occurred due to poor waste disposal [32]. These concerns have prompted protests and legal disputes against corporations operating in the MTP, advocating for more stringent regulations and accountability.

**Fig. 1:**
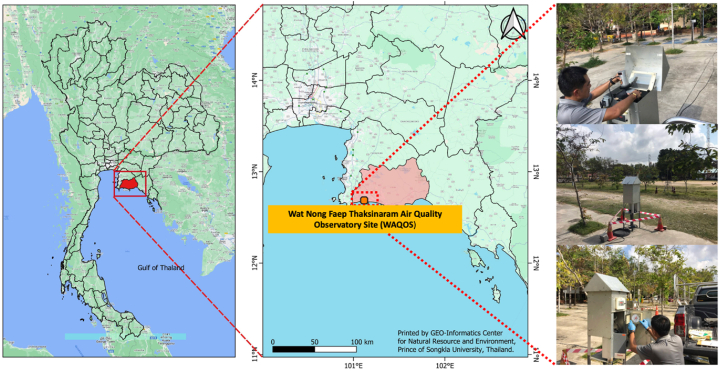
A map of Wat Nong Faep Thaksinaram Air Quality Observatory Site (WAQOS; 12.6862337 N 101.1154117 E) located adjacent to MTPIE in Rayong province, Thailand.

Total Suspended Particulate (TSP) samples in the vicinity of MTPIE were collected at Wat Nong Faep Thaksinaram Air Quality Observatory Site (WAQOS; 12.6862337 N 101.1154117 E), located approximately 1.7 km northwest from the MTPIE (see Fig. 1 and Fig. S1). Sampling for the continuous TSP was carried out for 24 h during each sampling interval, starting at 9:00 a.m. every day from February 14th to February 28th, 2023. The sampling technique was made easier by using a high-volume air sampler (Thermo Scientific: G3101) that operated at a constant flow rate of 40 ft^3^ min^−1^ (∼1.1 m^3^ min^−1^). TSP sampling was conducted without using the air pump of the high-volume air sampler for the first three days (February 11th to February 13th, 2023) and the last three days (March 1st to March 3rd, 2023) for field blank measurement. Quartz microfibre filters (QMFs) with a pore size of 2.2 μm, a thickness of 0.47 mm, and dimensions of 203 mm × 254 mm were used to collect all TSP samples. After collecting the samples, the filters were stored in a desiccator for 24 h before being weighed to avoid any possible contamination by water vapor. The weights of the filters before and after sampling were determined using a Mettler Toledo ME204 electronic balance. Simultaneously, climatic factors such as temperature and relative humidity were observed at each sampling location using a multi-parameter sensor (Bosch: BME280).

### 2.2 SR-ATR-FTIR analysis

The spectral data were acquired using an infrared micro-spectroscopy beamline called BL4.1 Infrared Spectroscopy and Imaging, which is located at the Synchrotron Light Research Institute (SLRI) in Nakhon Ratchasima, Thailand [10,33]. The spectra were obtained using an SR-FTIR (Hyperion 2000, Bruker Optics, Ettlingen, Germany) connected to an infrared microscope (Hyperion 2000, Bruker). The microscope was equipped with a 20 × ATR objective lens and a mercury cadmium telluride (MCT) detector cooled with liquid nitrogen. The measurement range covered wavelengths from 4000 to 800 cm^−1^. The measurements were performed using an ATR mode with a 20 × 20 μm^2^ aperture size, a spectral resolution of 4 cm^−1^, and 64 scans co-added. OPUS 7.5 (Bruker Optics Ltd., Ettlingen, Germany) was used to manage instrument management and spectrum acquisition. At WAQOS, a total of about 6 blank filter samples were collected, with three filters taken before sampling and three filters taken after sampling. Afterwards, SR-ATR-FTIR measurements were performed on all samples. These measurements were undertaken randomly 15 times at 15 different points on the filters. The results were then averaged to provide one spectrum per sample. The representative averaged spectrum of each sample was subsequently subtracted from the representative averaged spectrum of the blank filter to remove the background infrared spectrum from the target sample. The normalized spectra of TSP samples taken at WAQOS were then combined and used as the typical normalized spectrum of the aerosol samples. This study aimed to quantitatively identify water-soluble ionic species (WSIS) and organic functional groups (OFGs), such as aliphatic carbons (R-H), carbonyl species - hemicellulose - pectin - lectin (C=O), organo-nitrates (R-ONO_2_), aromatic nitro compounds (Arom-NO_2_), ammonium ions (NH_4_^+^), carbonate (CO_3_^2−^), nitrate ions (NO_3_^−^), sulfate species - sulfate ions - bisulfate ions (S=O), and calcium carbonate (CaCO_3_). In addition, the percentages of infrared absorption bands in the averaged representative spectra of TSP samples were computed with great precision using OPUS 7.5 software (Bruker Optics, Germany).

### 2.3 SR-XRF analysis

The elemental composition of TSP samples collected on filter papers underwent analysis utilizing the SR-XRF spectroscopy, which was employed at BL5.1W within the Siam Photon Source at the Synchrotron Light Research Institute (SLRI) [9]. The QMF sample was segmented into three sections, each approximately 1 × 1 cm in size, and affixed to the sample holder using Kapton tape. Positioned at a 45° angle to the incident X-ray photons, the sample holder facilitated exposure to a 15 keV monochromatic X-ray beam for a duration of 5 min, generated by Si(311) crystal-based X-ray tubes. Fluorescence photons emitted from the irradiated sample surface were captured by the Vortex EM silicon drift detector (SDD), situated 30 mm away and perpendicular to the synchrotron radiation beam. The Rigaku ZSX Primus IV wavelength dispersive X-ray fluorescence spectrometer was also utilized to quantify elemental components present in TSP filters. This non-destructive methodology integrates distinctive components within the spectrometer, including a scintillation counter (SC) detector sensitive to light elements and Gas Flow Proportional Counter (F-PC) detectors tailored for heavier elements (those exceeding atomic number 21). The device incorporates a hermetically sealed X-ray tube with a Rhodium target for stimulation, featuring an end window and a 20 mm^2^ aperture. Operating under vacuum conditions at ambient temperature, the X-ray tube functions at a power level of 4 kW. Analysis was conducted on TSP filters with a surface area of 2.5 cm^2^, with blank filter measurements incorporated, and adjustments for intensity were made to account for loaded filters. The parameter technique facilitated analysis, implemented via the ZSX software developed by Rigaku Corporation, Japan. In this study, 12 selected elements namely Manganese (Mn), Copper (Cu), Chromium (Cr), Zirconium (Zr), Phosphorus (P), Chlorine (Cl), Titanium (Ti), Iron (Fe), Magnesium (Mg), Sulfur (S), Aluminum (Al), and Calcium (Ca) was detected by SR-XRF. The measurements were executed under atmospheric conditions, with each filter paper sample subjected to three replicates. Subsequent spectral analysis encompassed energy calibration, peak identification, and fitting, conducted using PyMCA version 5.5.4 [34]. Relative elemental concentrations were determined based on the peak fit area of each observed element on the obtained spectra. Moreover, comprehensive statistical analyses were performed using IBM SPSS Statistics 23.

### 2.4 SR-XAS analysis

In this study, SR-XAS was conducted at SLRI in Thailand [35,36]. The synchrotron radiation produced by the storage ring, operating at a beam energy of 1.2 GeV, had a beam current that varied between 80 and 150 mA. The device emitted a photon flux with a peak value between 1.1 × 10^11^ and 1.7 × 10^11^ photons per second. The XANES and extended EXAFS were acquired at the Macromolecular Crystallography Beamline (BL7.2W), spanning an energy range from 5000 to 18000 eV. The Si(111) double crystal monochromator was employed to perform energy scanning within the designated analytical ranges. TSP filters were used, placed within a rectangular frame measuring 3 × 10 mm^2^. The calibration for Fe K-edge XAS was performed using a Fe foil at an energy of 7112 eV, utilizing a transmission mode. In addition, the oxidation states of the experimental samples were compared and clarified by examining fine powder of Fe_2_O_3_ (hematite), FeOOH (goethite), Fe_3_O_4_ (magnetite), and Fe_5_HO_8_∗4H_2_O (ferrihydrite) as standard samples. The EXAFS method is a powerful technique for determining the specific structural features around the atom being studied. It provides information about factors such as bond distance, coordination number, and chemical identity of the elements [37]. At the energy level associated with EXAFS, the photoelectrons that are emitted undergo excitation into a continuum that is mostly controlled by electron scattering, which is a crucial feature of the absorption phenomena. The movement of photoelectron waves, together with subsequent scattering events, creates interference patterns that depend on the distance traveled by these waves. Therefore, these interference patterns provide valuable information about the immediate structural environment surrounding the absorbing atom. The fundamental principles that govern EXAFS can be described using a mathematical formulation as illustrated in Equation (1) [38].

(1) $$x\left(k\right)=\sum_{j}\frac{N_{j}f_{j}\left(k\right)\exp\left[-2k^{2}\sigma_{j}^{2}\right]\;\exp\;\left[\frac{-2R_{j}}{\lambda }\right]}{kR_{j}^{2}}\;\sin\;[2kR_{j}+\delta_{j}\left(k\right)]$$

This formulation takes into account various factors, including the wave number (*k*) of the photoelectron, the number of neighboring atoms (*N*), the distance from the absorber atom (*R*), the current scattering factors (*f*(k) and *δ*(k)) of atoms near the excited atom, and the mean square displacement (*σ*^2^) in *R*. All of these factors collectively contribute to the EXAFS equation [38]. The EXAFS equation allows for the calculation of the number and proximity of nearby atoms, as well as the level of disorder within this spatial domain, by analyzing the scattering factors *f*(k) and *δ*(k) [39].

The EXAFS methodology provides a reliable foundation for understanding the complex structural details around the absorbing atom by utilizing electron scattering and interference events. EXAFS analysis utilizes mathematical formulations that incorporate important parameters, such as photoelectron wave number and scattering variables, to accurately determine nearby atoms, their spatial distribution, and the level of disorder present. This versatile analytical approach shows great potential in understanding the intricate structural dynamics found in many materials systems, therefore enhancing our understanding of fundamental chemical processes and enabling focused investigations in several scientific fields. In this study, the purpose of the EXAFS measurement was to determine the identity of the atoms in close proximity to the Fe atom.

The IFEFFIT program was employed for data processing and interpretation, incorporating the functionalities of ATHENA and ARTEMIS [40]. The XANES data was normalized and processed in both the pre-edge and post-edge regions. Linear combination fitting was conducted in the XANES region. The utilization of linear combination fitting (LCF) in the analysis of Fe EXAFS spectra involves a thorough evaluation of spectrum data obtained using X-ray absorption spectroscopy (XAS) to determine the proportional contributions of different iron species, particularly iron oxides, that are present in a given sample [41]. This analytical approach is especially valuable for determining the proportion of iron oxide in TSP samples, providing insights into their chemical composition. The process occurs in multiple crucial stages. First, Fe EXAFS spectra are obtained from the TSP samples using X-ray absorption spectroscopy techniques. This involves exposing the sample to X-rays and measuring the X-ray absorption at different energy levels. Therefore, it is necessary to have reference standards that consist of pure iron oxides (such as Fe_2_O_3_ and Fe_3_O_4_) as well as other compounds containing iron in order to carry out the linear combination fitting. The reference spectra act as standards for comparison, making it easier to identify the specific types of iron present in the TSP samples.

### 2.5 Enrichment factor computations

The idea of enrichment factor (*EF*) has been comprehensively employed to assess the impact of traffic releases [42,43], industrial exhausts [44], and other mining and/or ore processing [45] on aerosol chemical composition. In spite of the fact there is no certain rule for chosing the reference element, Fe, Al, and Si are regularly used for the calculations of *EF* [46]. For each selected element, Fe was applied as reference assuming minor impacts of pollutant Fe and the upper continental crustal composition [47]. The *EF* of an element *E* in a TSP sample can be explained as

(2) $$EF=\frac{{\left(E/R\right)}_{Air}}{{\left(E/R\right)}_{Crust}}$$

where *R* is a reference element. If *EF* approaches to one, crustal can be considered as the predominant emission source as illustrated in Equation (2). In addition, previous studies interpreted the extremely high *EF* values of some selected elements as consequences of anthropogenic activities [[48], [49], [50]].

### 2.6 Health risk assessment

Within the field of environmental health risk assessment, comprehending exposure routes is essential for assessing the potential health consequences of different substances. In order to assess the health risks associated with Mg, Al, P, S, Cl, Ca, Ti, Cr, Mn, Fe, Cu, and Zr in TSP samples, there are three exposure pathways of the average daily dose (ADD: mg kg^−1^ day^−1^) of selected elements, which can be described as follows: (*i*) Ingestion (ADD_ing_): Oral exposure is the route by which chemicals enter the body through the mouth and are absorbed into the bloodstream through the digestive system [51]. In the context of TSP samples, ingestion can occur when particles land on food, water, or surfaces that are subsequently consumed by humans. Assessing health hazards related with pollutants, such as heavy metals (e.g., Cr, Mn, Fe, Cu) and other elements found in TSP samples, is especially important when considering ingestion. After being consumed, these heavy metals have the ability to build up in the body gradually and may cause negative health consequences, which vary depending on the amount of exposure and the individual's vulnerability. (*ii*) Dermal Contact (ADD_derm_): This pathway refers to the direct contact between the skin and pollutants found in TSP samples [52]. When people encounter polluted surfaces, soil, dust, or water, certain toxins can be absorbed via the skin and enter the circulation. Although dermal contact may not be as significant as ingesting or inhalation in terms of exposure to TSP samples, it can nevertheless add to overall exposure levels, particularly for compounds like as metals (e.g., Al, Fe, Cu) that can attach to the skin or be present in dust particles. (*iii*) Inhalation (ADD_inh_): The inhalation route is the most direct and major channel for exposure to TSP samples [53]. This process entails the act of breathing in tiny particles suspended in the air that carry impurities. These particles can settle in the respiratory system and potentially enter the bloodstream. TSP samples frequently contain tiny particulate materials, such as metals (e.g., Al, Cr, Fe) and other elements, which can be released into the air by various processes, such as industrial activity, combustion, or natural sources (e.g., dust storms). Exposure to these particles by inhalation can result in respiratory complications, cardiovascular disorders, and other detrimental health consequences, especially when the exposure persists for a prolonged duration [54].

In this study, three distinct models were utilized to compute human exposure to particulate metals. These models calculate the ADD (mg kg^−1^ day^−1^) of selected elements through ingestion (ADD_ing_), dermal contact (ADD_derm_), and inhalation (ADD_inh_) as exposure pathways. These calculations can be performed using Equations (3), (4), (5).

(3) $${ADD}_{ing}=\frac{c\times R_{ing}\times CF\times EF\times ED}{BW\times AT}$$

(4) $${ADD}_{derm}=\frac{c\times SA\times CF\times SL\times ABS\times EF\times ED}{BW\times AT}$$

(5) $${ADD}_{inh}=\frac{c\times R_{\mathrm{int}}\times EF\times ED}{PEF\times BW\times AT}$$

The variables ADD_ing_, ADD_derm_, and ADD_inh_ represent the daily quantity of selected elements that are ingested, come into touch with the skin, and are inhaled, respectively. These amounts are measured in milligrams per kilogram per day (mg kg^−1^ d^−1^). These three models are derived from the Exposure Factors Handbook put forward by the United States Environmental Protection Agency [55]. Furthermore, the components that contribute to the amount of exposure in dosage models can be stated as follows: *C* represents the concentration of the chosen metals in TSP, measured in micrograms per gram (μg g^−1^). *R*_ing_: The rate at which soil is consumed, which measured in milligrams per day (mg d^−1^), is 100 for adults [56]. *EF* refers to the frequency of exposure, measured in days per year. For adults, the exposure frequency is 350 days per year (d y^−1^) [57]. *ED* refers to the period of exposure, measured in years. For humans, the standard duration is 24 years. *BW* refers to the average body weight in kilograms (kg), which is 55.9 for adults [57]. *AT* refers to the average time in days, calculated by multiplying 365 by the *ED* (Effective Dose) [56]. *CF* refers to the conversion factor, which is expressed in kilograms per milligram (kg mg^−1^) and has a value of 1 × 10^−6^ [57]. *R*_inh_: The rate at which air is inhaled, measured in cubic meters per day (m^3^ d^−1^). The rate for adults is 20 m^3^ d^−1^m [57]. The particle emission factor (*PEF*) is a measure of the amount of particles emitted per unit mass, expressed in cubic meters per kilogram (m^3^ kg^−1^). The specific value of *PEF* is 1.32 × 109 [57]. The *SA* refers to the surface area of the skin that comes into contact with dust particles. For adults, this surface area is estimated to be 5000 cm^2^ [57]. SL refers to the skin adhesion factor for dust, measured in milligrams per square centimeter (mg cm^−2^) [57]. ABS is the dermal absorption factor, unique to metals, with a value of 0.001 [57]. It is important to underline that the variability in the potential for toxicity due to uncertainties in input parameters. It is also crucial to acknowledge that various uncertainties can significantly alter the projected values of ADD_ing_, ADD_derm_, and ADD_inh_. An earlier study reported that the gravimetric analysis of TSP might have both positive and negative artifacts, which may introduce biases with systematic seasonal patterns [58]. According to a prior study [59], healthy persons with normal weight had inhalation rates that were 6–21 % greater than those who were overweight or obese. Infants and children aged 3 weeks to less than 7 years breath 1.6 to 4.3 times more air than adults aged 23–96 years [59].

## 3 Results & discussion

### 3.1 SR-ATR-FTIR spectra and percentage contributions of OFGs

In recent decades, numerous investigations have been carried out to elucidate the chemical compositions of aerosols as a means of identifying their potential sources and allocating them accordingly [[5], [6], [7], [8], [9], [10]]. A previous study used SR-ATR-FTIR to analyze PM_10_ collected in ambient air around the Lamchabang Seaport, Thailand [10]. They identified various orgnanic functional groups in these particles, with sulfates and aliphatic carbons being the most abundant. The amount of sulfate varied depending on the location, with the port area having the highest concentration. The study suggests that shipping activities are a major source of aliphatic carbons, organo-nitrates, and aromatic nitro compounds found in the air, while sulfates and ammonium likely come from sea salt aerosols and fertilizer dust, respectively [10]. ATR-FTIR was also employed to chemically characterize PM_1.0_ samples in the ambient air near Phoenix, Arizona [11]. One-third of these particles were organic and included biogenic materials, oxygenated compounds, and hydrocarbons. The researchers identified three main sources of these particles: local plants, urban emissions from Phoenix, and a regional background source. Phoenix emissions had the greatest impact, creating both fresh and secondary particles in the air [11].

**Fig. 2:**
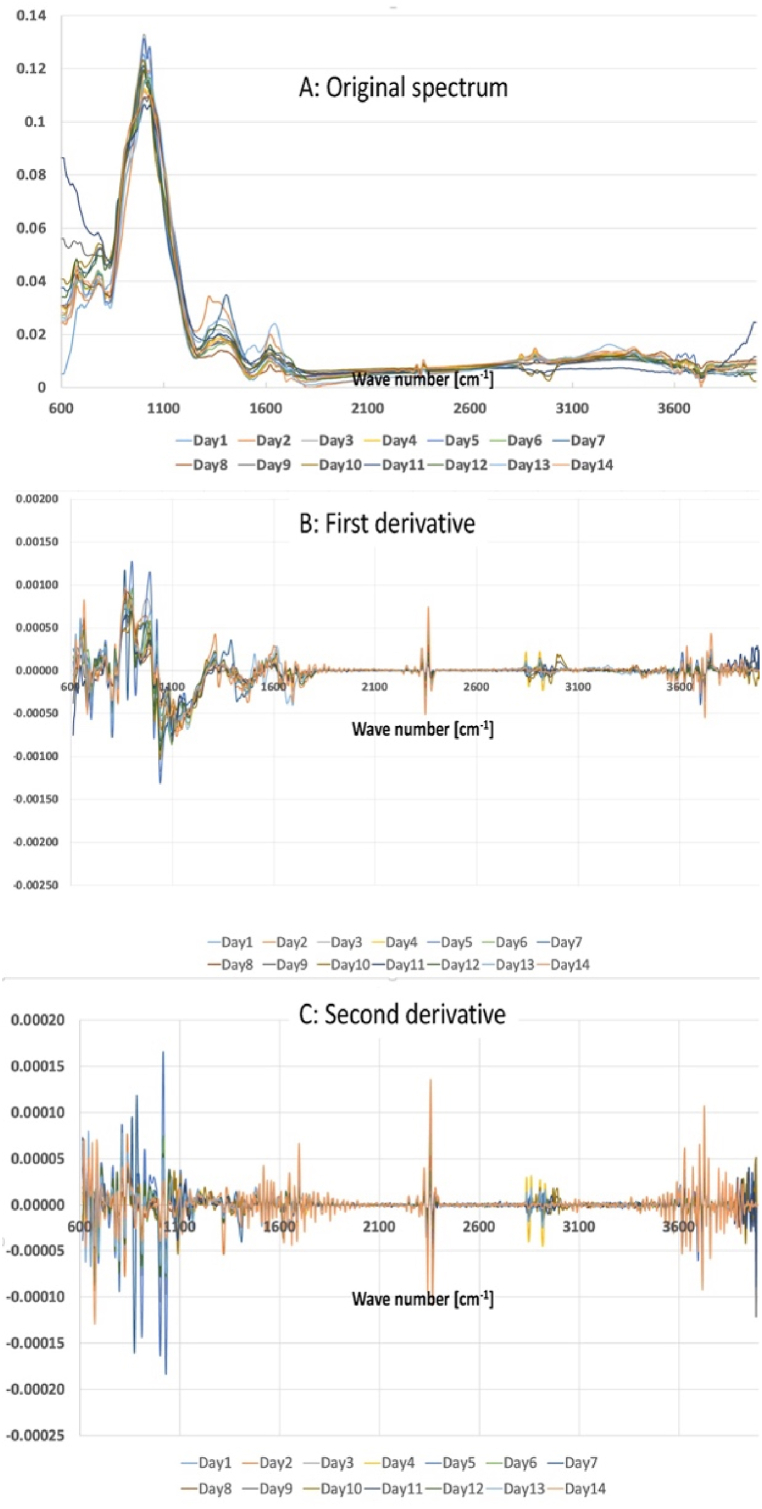
Three different ATR-FTIR spectra of TSP collected at WAQOS namely (a) original spectrum, (b) first derivative, and (c) second derivative.

An examination was carried out to compare the 14 SR-ATR-FTIR spectra of TSP samples. The results of this inquiry are shown in Fig. 2(A–C). It is crucial to emphasize that all SR-ATR-FTIR spectra of the TSP samples underwent subtraction from the spectra of the QMF (Quartz Microfiber) field blanks. The similarity observed in the features of all SR-ATR-FTIR spectra can be attributed to the presence of similar distributions of OFGs across the 14 TSP samples. OFGs cause distinct bond absorptions at specific positions and intensities within the infrared (IR) spectrum. Despite possible little variations among the 14 SR-ATR-FTIR spectra, four distinct features were consistently found in all absorption bands. The most significant absorbances were seen in the wavenumber range of 995∼1033 cm^−1^, indicating the presence of alkene functionalities (such as C=C bending), anhydride moieties (such as CO-O-CO stretching), and sulfoxide groups (such as S=O stretching). Additionally, two significant absorption peaks were detected in the wave number range of 1200–1700 cm^−1^, suggesting the presence of sulfonic acid, sulfonamide, and sulfonate groups (such as S=O stretching) as well as unsaturated ketones, alkenes, and cyclic alkenes (such as C=C stretching) in the TSP samples collected at the WAQOS. Furthermore, two small absorption peaks were seen within the wave number range of 2850–2917 cm^−1^, indicating the presence of amine salts (such as N-H stretching) based on their distinctive absorbance bands. Finally, a wide peak covering the wavenumber range of 3100–3600 cm^−1^ indicates the existence of exchangeable protons, mainly derived from alcohol, amine, amide, or carboxylic acid groups. Therefore, it is logical to conclude that all OFGs found in the TSP samples have comparable spectrum properties.

**Fig. 3:**
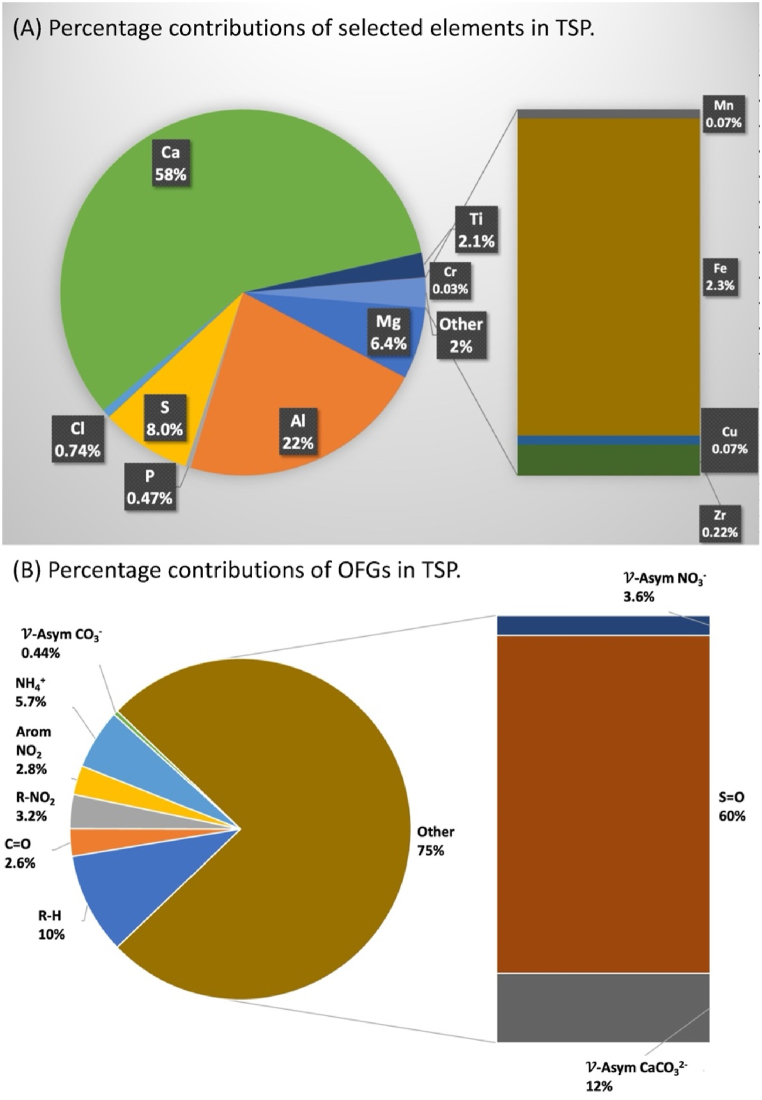
Percentage contributions of selected metals (a) and OFGs (b) in TSP collected at WAQOS.

Fig. 3(A–B) display the distribution of particulate OFGs among a group of 14 samples, as measured at WAQOS. The main components of the water-soluble ionic species (WSIS) in the composition were primarily sulfate species, including sulfonyl chloride, sulfonate, sulfonamide, sulfonic acid, and sulfone compositions. These sulfate species accounted for 65 % of the overall composition of the WSIS. Additionally, calcium carbonate (CaCO_3_) was identified as the second most prevalent organic fossil fuel, accounting for 12 % of the total composition. One notable finding among the reported OFGs was the detection of asymmetric stretching vibrations associated with OH groups. These vibrations are typically found in alcohols and carboxylic acids. The OH group was the third largest OFG group, contributing 10 %. In addition, the analysis of the TSP samples taken at WAQOS revealed a significant presence of ammonium ions (NH_4_^+^), accounting for 5.7 % of the total composition. The notable presence of organo-nitrates, making up 3.2 % of the overall OFG composition, is of importance, as well as the presence of aromatic nitro compounds, which account for 2.8 %. These data indicate that fossil fuel burning from industrial operations has a substantial impact on emissions.

### 3.2 Percentage contribution of selected elements as assessed by SR-XRF

Multiple studies have methodically examined the elemental compositions of aerosols in order to evaluate the emission intensities of sources of pollutants [9,12,13]. Nevertheless, there is still a requirement for inventive analytical approaches to enhance the understanding of data. This study utilizes SR-XRF spectroscopy to accurately identify the chemical composition of industrial emission aerosols. The average proportion of components in the TSP samples is carefully examined and compared (See Fig. 3(A–B)). The atmospheric percentage allocations of 13 elemental species were determined using a complete dataset consisting of 14 samples taken at the WAQOS from 14th to 28th of February 2023, as shown in Fig. 3(A–B). Since all TSP samples were collected using Quartz Microfiber Filters (QMFs), the SR-XRF spectra of the aerosol specimens are compared to those obtained from the field blanks for differential analysis.

The percentage contributions of selected elements offer valuable insights into the dynamics of local air quality and the possible impact of industrial activity. The high abundance of Ca, accounting for 58 % of the TSP composition, indicates that it likely originates from many sources, such as dust from nearby building activities or naturally occurring deposits of limestone or calcite minerals [60,61]. Al is another major contributor, accounting for 22 % of the emissions. This suggests that it may be coming from industrial activities or the spread of mineral dust nearby [62,63]. Sulfur, comprising 8.0 % of the TSP, may be associated with activities such as the burning of fossil fuels or industrial processes that release sulfur oxides into the atmosphere [64]. Furthermore, the presence of Mg (6.4 %) and Ti (2.1 %) can be attributed to crustal minerals or soil dust, thereby adding to the total elemental makeup [80]. Cl, P, Cr, Mn, Fe, Cu, and Zr are all present in small amounts, ranging from 0.03 % to 0.74 %. These elements can be used as indicators of certain industrial activities or as remnants of natural processes [81,82]. A thorough analysis is required to gain a deeper understanding of their origins, which involves studying the local industrial profiles and geological properties of the region. The importance of Ca and Al highlights the role of industrial operations or natural processes involving these metals in influencing the composition of TSP samples. Thorough study is necessary when S is detected, as it indicates probable pollution sources, such as coal-fired power stations or industrial sites that use sulfur-based compounds. Likewise, the relatively small amounts of Cr, Mn, Cu, and Fe require careful examination to determine their sources and impact on the air quality in the area. In the future, it is important to conduct study on the size distribution of TSP particles, compare data from multiple sample times to understand seasonal fluctuations, and investigate potential health hazards related with detected elements. To get a more thorough comprehension of air quality dynamics near the MTPIE, it is necessary to include more data and broaden the area of study. This will enable the development of well-informed policies for pollution control and environmental preservation in the region.

### 3.3 Percentage contribution of iron oxides as assessed by EXAFS

**Fig. 4:**
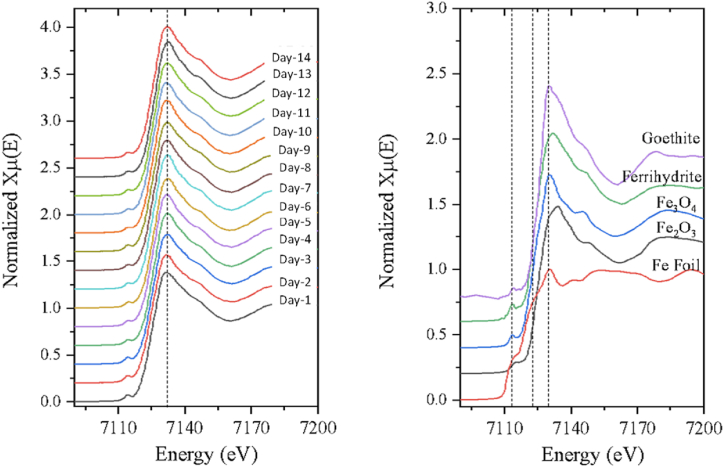
Iron oxidation states of TSP collected at WAQOS as assessed by EXAFS.

In this study, the obtained Fe EXAFS spectra from the TSP samples are compared to the reference spectra using a fitting technique (see Fig. 4). Table 1 displays the outcomes of linear combination fitting, namely the percentage weight content, of Fe EXAFS spectra acquired from TSP samples. The table presents the proportionate contributions of several iron species (i.e. Fe_2_O_3_, FeOOH, Fe_3_O_4_, and Fe_5_HO_8_∗4H_2_O), in each sample during a period of 14 consecutive days. After careful investigation, it is clear that the compositions of iron species in the TSP samples show significant change during the sampling period. The abundance of each iron species varies significantly on a daily basis, indicating that there are dynamic alterations in environmental circumstances or anthropogenic activities that affect the chemical composition of particulate matter. On the first day, the most abundant iron species in the sample is magnetite, making up 35.5 % of the sample. This is followed by ferrihydrite, hematite, and goethite with the percentage contribution of 25.0 %, 19.8 % and 19.7 %, respectively. However, on the sixth day, there is a noticeable change, as hematite makes up 44.1 % of the sample with the absence of ferrihydrite, whereas goethite and magnetite show decreased proportions. The variety in iron species distribution within TSP samples highlights the altering nature of this distribution and shows the possible impact of environmental conditions on the composition of particulate matter.

**Table 1:** Linear combination fitting (% weight content) of Fe EXAFS spectra of 14 TSP samples.

Sample	Fe_2_O_3_	FeOOH	Fe_3_O_4_	Fe_5_HO_8_∗4H_2_O
	(hematite)	(goethite)	(magnetite)	(ferrihydrite)
Day1	19.8	19.7	35.5	25.0
Day2	16.1	12.1	49.4	22.4
Day3	21.2	21.7	38.7	18.5
Day4	21.6	35.6	39.5	3.34
Day5	17.5	34.8	46.7	0.98
Day6	44.1	26.4	29.6	0.00
Day7	7.92	35.9	50.0	6.19
Day8	34.0	30.4	35.6	0.00
Day9	22.6	21.5	39.3	16.7
Day10	18.0	36.9	45.1	0.00
Day11	27.0	34.2	38.8	0.00
Day12	24.1	38.0	34.1	3.82
Day13	17.2	41.4	41.4	0.00
Day14	34.2	33.8	32.0	0.00

In spite of the fact that the percentage contribution of iron species fluctuated considerably during the monitoring interval, magnetite showed the greatest contribution followed by goethite, hematite, and ferrihydrite with the average values of 39.7 ± 6.27 %, 30.2 ± 8.53 %, 23.2 ± 9.14 %, and 6.90 ± 9.37 %, respectively. The findings are in good agreement with a previous study using EXAFS for determining iron species in PM_10_ and PM_2.5_ collected at an iron and steel industrial district in Shanghai, China [83]. The comparative contents of Fe_3_O_4_ in the air samples of iron and steel industrial district are greater than those of the other two districts (i.e. downtown and suburban), possible because the Fe_3_O_4_ is one of the raw materials in the iron and steel industry [83]. Table 1 offer significant insights into the temporal fluctuations of iron species composition in TSP samples, elucidating the probable sources and mechanisms that influence the dynamics of particulate matter in the studied environment. Conducting additional research on the fundamental causes behind these changes could improve our comprehension of the dynamics of air quality and provide insights for developing methods to reduce particulate matter pollution.

### 3.4 Hierarchical cluster analysis (HCA)

**Fig. 5:**
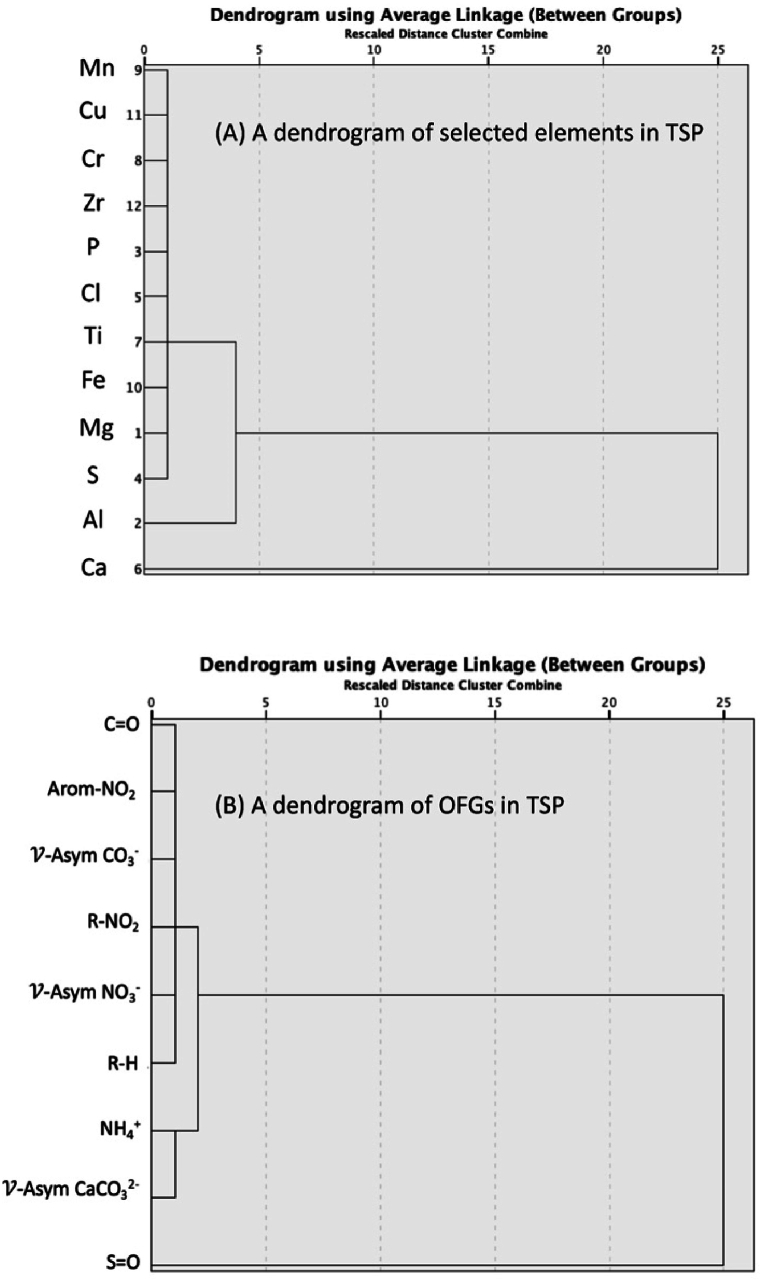
A dendrogram of selected metals (a) and OFGs (b) in TSP collected at WAQOS as assessed by EXAFS and ATR-FTIR.

The agglomerative hierarchical clustering algorithm (HCA) was used to create a dendrogram of selected elements and organic functional groups (OFGs) in TSP samples. This was done by analyzing the % chemical compositions from both the SR-XRF and the SR-ATR-FTIR spectra. This analysis examines the fundamental connections within TSP gathered from the WAQOS at the MTPIE in Rayong Province, Thailand. The study employs a dendrogram, which is a diagram that shows branching, to reveal two main groups of elements. This provides insight into their possible origins and relationships. The dendrogram divides the analyzed items into two main clusters: Cluster 1 and Cluster 2. Cluster 1 can be further divided into Sub-cluster 1a, which includes elements like Mn, Cu, Cr, Zr, P, Cl, Ti, Fe, Mg, and S (see Fig. 5(A–B)). These elements share similarities in their abundance or probable sources within the TSP. In contrast, Sub-cluster 1b exclusively comprises Al, indicating possible variations in origin or behavior when compared to its counterparts in Sub-cluster 1a. Cluster 2 exclusively consists of Ca, suggesting a unique origin or special geochemical characteristics that differentiate it from other elements.

The arrangement of components in Sub-cluster 1a of Cluster 1 suggests a common source or similar chemical properties, maybe originating from industrial activity, soil dust, or a combination of both. The detection of Mn, Cu, Cr, and Fe suggests that there may be contributions from metal processing or combustion operations within MTPIE [84]. On the other hand, the presence of P and S might be used as indications for activities involving fertilizers or the combustion of fossil fuels [64,65]. Al in Sub-cluster 1b shows a correlation with elements in Sub-cluster 1a, although it has different features, indicating potential connections with mineral dust or specialized industrial pollutants [66]. Ca in Cluster 2 is different from other elements, indicating a distinct origin that may be linked to natural geological sources such as limestone or minerals rich in calcite that are commonly found in the surrounding environment. Construction operations may also contribute to the occurrence of Ca within the TSP matter [67]. However, the dendrogram offers insights into the connections between elements based on their abundance rather than exact sources. Therefore, additional research is needed to determine the exact origins.

Further analysis was performed by using HCA of OFGs in TSP samples. The first main cluster bifurcates into two sub-clusters, each comprising distinct OFGs. Sub-cluster 1a encompasses several OFGs, including C=O, NO_2_ asymmetric stretching, *ν*-asym CO_3_^2−^, NO_2_ asymmetric stretching (R-ONO_2_), *ν*-asym NO_3_^−^, and R-H. These OFGs likely originate from diverse anthropogenic and biogenic sources, such as vehicle emissions, industrial activities, and biomass burning. The presence of functional groups associated with nitrate (NO_3_^−^) and carbonate (CO_3_^2−^) suggests contributions from combustion processes and secondary aerosol formation in the atmosphere, respectively. Furthermore, the detection of C=O and C-H functional groups indicates the presence of organic compounds derived from various sources, including fossil fuel combustion and biogenic emissions. In contrast, Sub-cluster 1b of the first main cluster comprises *ν*-asym CaCO_3_^2−^ and NH_4_^+^. The presence of these OFGs suggests a potential association with mineral dust and ammonium compounds. Calcium carbonate (CaCO_3_) is commonly found in dust particles originating from natural sources such as soil erosion and geological activities. The detection of ammonium (NH_4_^+^) functional groups may indicate contributions from agricultural activities, industrial emissions, or atmospheric reactions involving ammonia.

The second main cluster comprises OFGs related to sulfur-containing compounds, including S=O stretching, -SO_4_^-^, and -HSO_4_^-^. These OFGs are characteristic of sulfate aerosols, which often arise from the oxidation of sulfur dioxide (SO_2_) emitted from industrial processes, fossil fuel combustion, and biomass burning. Sulfate aerosols play a significant role in atmospheric chemistry, influencing air quality, climate, and human health. Overall, the dendrogram analysis of both selected elements and OFGs in TSP collected at WAQOS near MTPIE provides valuable insights into the sources and composition of both inorganic and organic aerosols in the region. The presence of diverse OFGs associated with anthropogenic, biogenic, and mineral dust sources underscores the complexity of aerosol composition and the interconnectedness of regional air quality dynamics. These findings contribute to our understanding of atmospheric processes and pollutant sources, facilitating informed decision-making for air quality management and environmental protection efforts in industrialized regions like MTPIE. In the future, it is crucial to conduct research on correlating specific components with industrial activity in the MTPIE, comparing dendrogram data with local geological knowledge, and evaluating potential health concerns connected with elements in each cluster. By combining dendrogram analysis with additional data, a more detailed understanding of the elemental composition of TSP in the WAQOS may be achieved. This will help in developing informed approaches for reducing air pollution, identifying pollution sources, and preserving the environment around the MTPIE.

### 3.5 Enrichment factors of selected elements

**Fig. 6:**
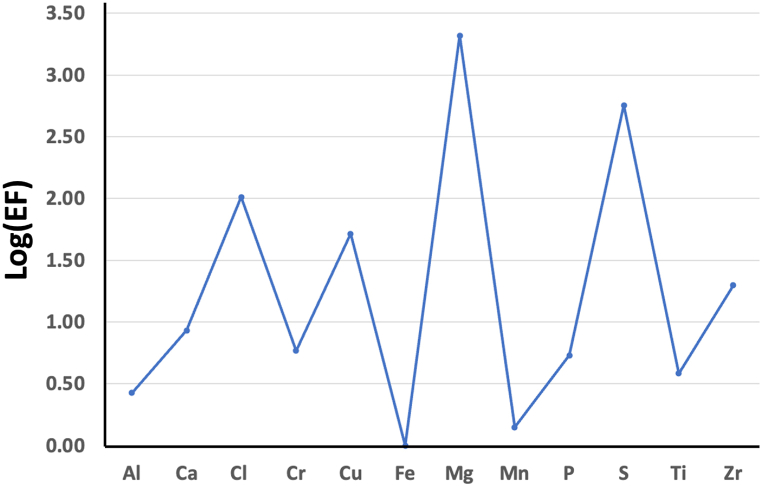
Log (EF) of selected metals in TSP collected at WAQOS as assessed by EXAFS.

The logarithmic enrichment factors (*EFs*) of the 12 chosen elements in TSP samples recorded at WAQOS from February 14th to February 28th, 2023, are shown in Fig. 6. The elements are listed in descending order as follows: Mg > S > Cl > Cu > Zr > Ca > Cr > P > Ti > Al > Mn > Fe. The results can be categorized into three groups using an arbitrary scale, which is based on earlier studies [42,68]. Firstly, the elements Mg and S exhibited significant enrichment, with a Log(*EF*) ranging from 2 to 3.5. Furthermore, there was a significant enrichment of Cl, Cu, and Zr, with Log(*EF*) ranging from 1 to 2. Furthermore, Al, Ca, Cr, Fe, Mn, P, and Ti did not see an increase in Log(*EF*) (i.e., Log(*EF*) < 1). It is crucial to emphasize that over 58 % of the Log(*EF*) values were less than 1, just 16 % were greater than 2, and 26 % were less than 1. The Log(*EF*) values can be significantly altered by various factors, including traffic exhaust, construction activities, crustal releases, industrial emissions, biomass burnings, re-suspended particles, and sea spray particles [42,43,[48], [49], [50]]. Given that the Log(*EF*) values of Mn approached zero, indicating *EF* values close to one, it may be inferred that the crustal dust is a likely source of this element. The relatively low Log(*EF*) values of aluminum reported in this study are consistent with a prior study [69]. The most plausible explanation for the extremely low Log(*EF*) values observed in WAQOS is that terrestrial soil is releasing particulate Al. Although previous research highlighted Mg as a reliable signal of firework displays [70,71], the current data indicate that Mg may not be a suitable indicator for this source. A prior investigation evaluated the air quality in Raipur, India, a city characterized by significant industrial operations and coal-fired power plants. The study found a notable presence of MgSO_4_ in coarse particles [72]. Another study was carried out in Jinzhong city, a renowned industrial city in China, emphasizing the significance of MgSO_4_ as the primary sulfate salt in TSP [73]. The remarkably elevated Log(*EF*) values of Mg and S can be attributed to industrial operations and are consistent with previous research [72].

### 3.6 Health risk assessment of selected elements

This section investigated the health hazards linked to the presence of substances found in 14 TSP samples, evaluating the daily consumption from ingestion, inhalation, and contact with the skin (see Table 2). The average daily dose (ADD) in milligrams per kilogram of body weight per day (mg kg^−1^ day^−1^) for both adults and children provides insight into three possible exposure pathways namely ingestion (ADD_ing_), dermal contact (ADD_derm_), and inhalation (ADD_inh_). Ca had the greatest ADD among all exposure routes, which is likely due to its substantial presence in soil and crustal materials. In contrast, elements such as Mg, S, Fe, and Ti exhibited considerably daily intake levels, suggesting a combination of natural and human-induced sources affecting their existence. Al demonstrated comparatively high ADD values, particularly when ingested, which raises concerns about potential neurological effects linked to long-term exposure to high doses of this heavy metal. Elements such as Mn, Cr, Cu, and Zr, although found in smaller amounts, deserve consideration because of their harmful effects on health, even at low levels of exposure [74,75].

**Table 2:** Statistical descriptions of the average daily dose (mg kg^−1^ day^−1^, ADD) of selected elements in TSP via inhalation (inh), ingestion (ing), and dermal contact (derm).

Adults	ADD_ing_		ADD_derm_		ADD_inh_		Children	ADD_ing_		ADD_derm_		ADD_inh_	
(mg kg^−1^ day^−1^)	Aver	Stdev	Aver	Stdev	Aver	Stdev	(mg kg^−1^ day^−1^)	Aver	Stdev	Aver	Stdev	Aver	Stdev
Mg	4.01E-01	2.09E-01	1.13E-02	5.88E-03	8.01E-02	4.18E-02	Mg	5.83E-01	3.04E-01	1.26E-03	6.57E-04	1.46E-02	7.60E-03
Al	1.38E+00	6.54E-01	3.89E-02	1.84E-02	2.77E-01	1.31E-01	Al	2.01E+00	9.53E-01	4.35E-03	2.06E-03	5.03E-02	2.38E-02
P	2.98E-02	7.77E-03	8.37E-04	2.19E-04	5.95E-03	1.55E-03	P	4.33E-02	1.13E-02	9.36E-05	2.44E-05	1.08E-03	2.83E-04
S	5.04E-01	1.18E-01	1.42E-02	3.33E-03	1.01E-01	2.37E-02	S	7.34E-01	1.72E-01	1.59E-03	3.73E-04	1.84E-02	4.31E-03
Cl	4.68E-02	3.38E-02	1.32E-03	9.51E-04	9.37E-03	6.76E-03	Cl	6.82E-02	4.92E-02	1.47E-04	1.06E-04	1.71E-03	1.23E-03
Ca	3.64E+00	2.25E+00	1.02E-01	6.33E-02	7.27E-01	4.50E-01	Ca	5.29E+00	3.28E+00	1.14E-02	7.07E-03	1.32E-01	8.19E-02
Ti	1.35E-01	8.23E-02	3.79E-03	2.32E-03	2.70E-02	1.65E-02	Ti	1.96E-01	1.20E-01	4.24E-04	2.59E-04	4.91E-03	3.00E-03
Cr	1.74E-03	1.51E-03	4.89E-05	4.24E-05	3.48E-04	3.01E-04	Cr	2.53E-03	2.19E-03	5.47E-06	4.74E-06	6.33E-05	5.49E-05
Mn	4.15E-03	1.33E-03	1.17E-04	3.73E-05	8.31E-04	2.65E-04	Mn	6.05E-03	1.93E-03	1.31E-05	4.17E-06	1.51E-04	4.82E-05
Fe	1.45E-01	3.56E-02	4.09E-03	1.00E-03	2.90E-02	7.11E-03	Fe	2.11E-01	5.18E-02	4.57E-04	1.12E-04	5.29E-03	1.29E-03
Cu	4.35E-03	1.98E-03	1.23E-04	5.58E-05	8.71E-04	3.96E-04	Cu	6.34E-03	2.89E-03	1.37E-05	6.23E-06	1.58E-04	7.22E-05
Zr	1.40E-02	7.69E-03	3.94E-04	2.16E-04	2.80E-03	1.54E-03	Zr	2.04E-02	1.12E-02	4.40E-05	2.42E-05	5.09E-04	2.80E-04

As illustrated in Table 2, TSP contain potentially hazardous components that can be inhaled and ingested by children, who are especially vulnerable to these toxic elements. The fact that children's lungs are smaller and their metabolic rates are faster than those of adults means that they take in more air per kilogram of body weight than adults do [76]. This corresponds to a higher consumption of TSP, which contains heavy metals and other contaminants. Because of their inherent curiosity, infants frequently engage in hand-to-mouth exploration, which can result in their unintentionally consuming dust and dirt particles that are loaded with these chemical contaminants [77,78]. This further increases their exposure. Children are further exposed to these toxins when they play on the ground, which is a place where dust tends to accumulate and concentrate [79]. Because of the combination of these characteristics, which include faster inhalation rates and behavior that involves hand-to-mouth contact, children absorb a much greater quantity of heavy metals and elements from TSP in comparison to adults. As a consequence, this emphasizes the significance of taking into account age-specific susceptibilities in health risk evaluations related to TSP exposure. Although ADD values provide valuable information, a thorough health risk evaluation requires a comparison with recognized safety thresholds for each element. Heightened concentrations of Al, Cr, and Mn emphasize the importance of careful surveillance and implementation of measures to reduce potential health hazards. The study's dependence on approximated exposure pathways emphasizes the necessity for additional research on real exposure scenarios, taking into account individual behaviors and microenvironments. Potential areas for future research involve performing comprehensive health risk assessments by comparing ADD values with established safety thresholds, determining the exact sources of pollution through source apportionment studies, and enhancing risk assessments by considering individual exposure patterns to achieve more precise risk evaluations.

## 4 Conclusions

This study presents a thorough analysis of TSP collected from the MTPIE area, employing advanced synchrotron-based techniques such as ATR-FTIR, EXAFS, and XRF. The findings revealed that magnetite was the dominant iron oxide species in TSP, accounting for an average of 39.7 % of the total iron content, followed by goethite at 30.2 %, hematite at 23.2 %, and ferrihydrite at 6.9 %. These variations in iron species composition suggest that changes in environmental conditions or anthropogenic activities significantly influence particulate matter. The study also identified that sulfate species and calcium carbonate were major components of the WSIS in TSP, contributing 65 % and 12 %, respectively, to the overall composition. The presence of organo-nitrates (3.2 %) and aromatic nitro compounds (2.8 %) further underscores the substantial impact of industrial emissions in the region. HCA showed that elements such as Mn, Cu, Cr, Zr, and Ti, along with specific organic functional groups, clustered together, suggesting common sources, likely from industrial activities and soil dust.

Furthermore, the EF analysis revealed that magnesium (Log(EF) of 2.5) and sulfur (Log(EF) of 2.3) were significantly enriched, pointing to industrial processes as the primary sources. The health risk assessment highlighted the higher ADD of calcium through ingestion (4.2 mg kg⁻^1^ day⁻^1^) and inhalation (2.1 mg kg⁻^1^ day⁻^1^), particularly affecting children due to their higher inhalation rates and behavior patterns. This study emphasizes the need for age-specific health risk assessments and demonstrates the critical role of synchrotron-based techniques in enhancing our understanding of aerosol composition and guiding pollution management strategies in industrial areas like MTPIE.

## CRediT authorship contribution statement

**Siwatt Pongpiachan:** Writing – review & editing, Writing – original draft, Formal analysis, Data curation, Conceptualization. **Kanjana Thumanu:** Resources, Methodology, Investigation. **Waraporn Tanthanuch:** Methodology, Investigation. **Duangjai Srisamut:** Software, Formal analysis, Data curation. **Jureerat Pradabsri:** Visualization, Validation, Supervision, Software. **Muhammad Zaffar Hashmi:** Visualization, Validation, Supervision. **Yan Sun:** Visualization, Validation, Supervision. **Saran Poshyachinda:** Visualization, Validation, Resources.

## Data and code availability statement

Data will be made available on request.

## Funding

This research has received funding support from the NSRF via the Program Management Unit for Human Resources & Institutional Development, Research and Innovation [grant number B11F670110].

## Declaration of competing interest

The authors declare that they have no known competing financial interests or personal relationships that could have appeared to influence the work reported in this paper.
