# Supplementary material for: Using synchrotron based ATR-FTIR, EXAFS, and XRF to characterize the chemical compositions of TSP in industrial estate area
Source: Heliyon. 2024 Oct 12;10(20):e39215. doi: 10.1016/j.heliyon.2024.e39215 (PMC11530839; doi:10.1016/j.heliyon.2024.e39215)
Supplement: Multimedia component 1 [file mmc1.docx]

| 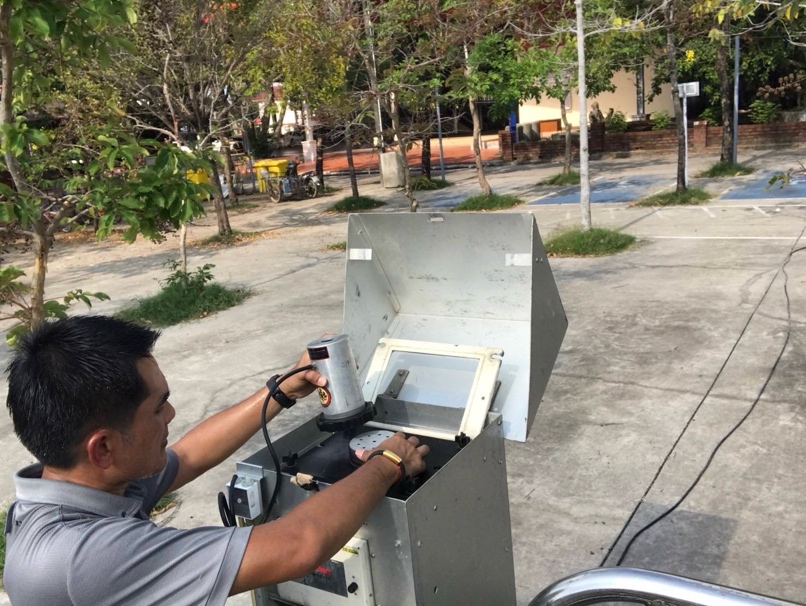 | 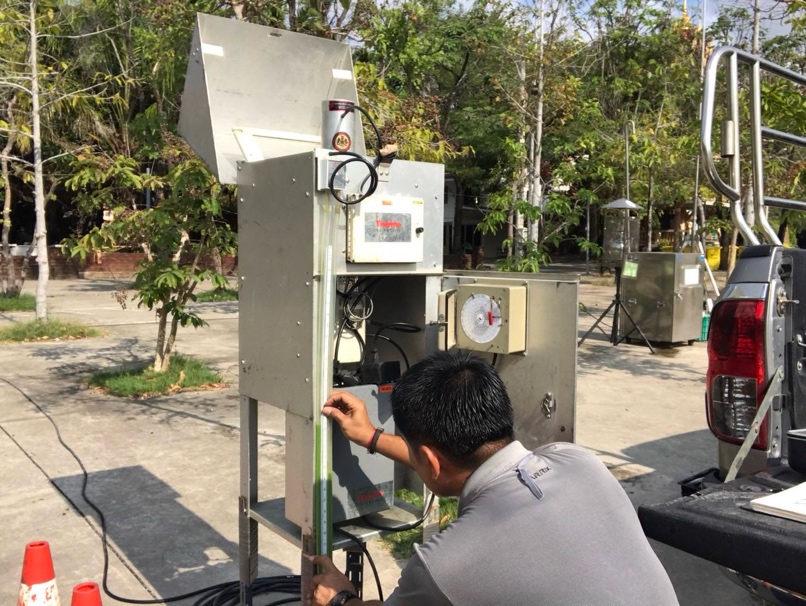 |
| --- | --- |
| 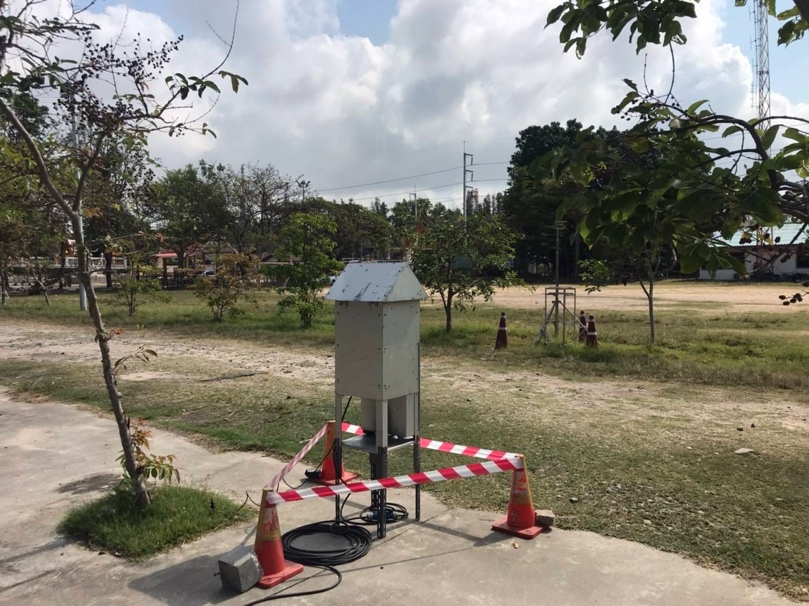 | 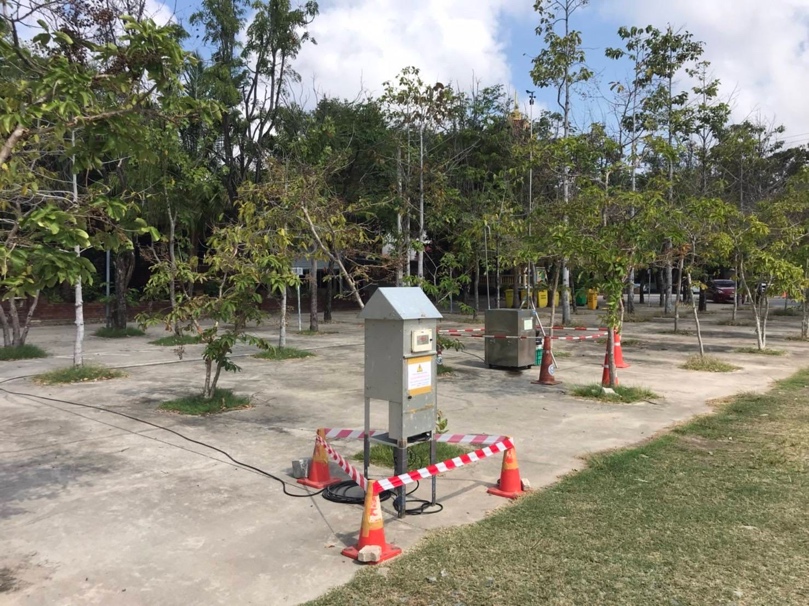 |
| 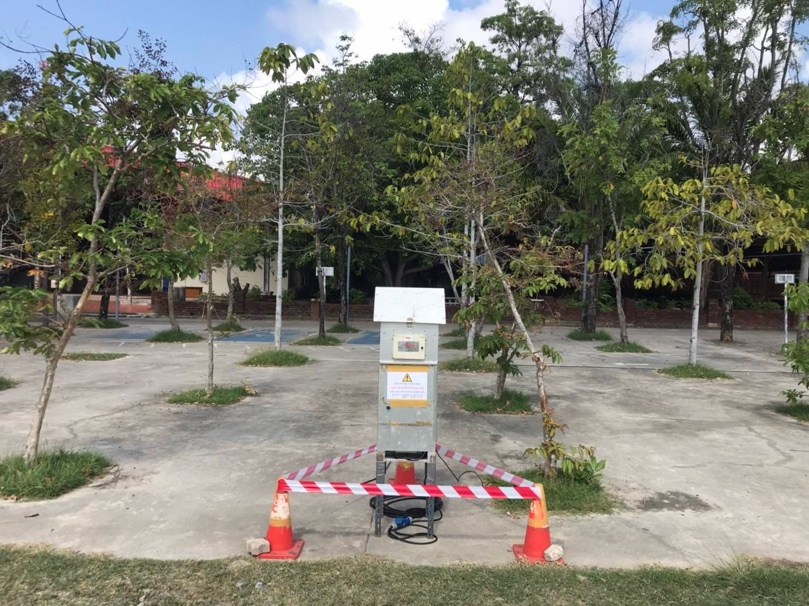 | 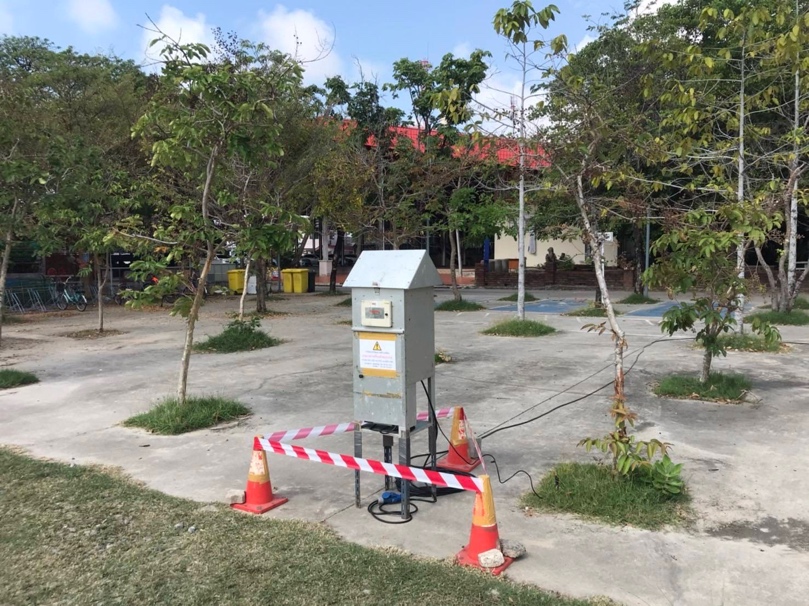 |

Fig. S1. The sampling position of HVAS at WAGOS from February 14^th^ to February 28^th^, 2023.

| 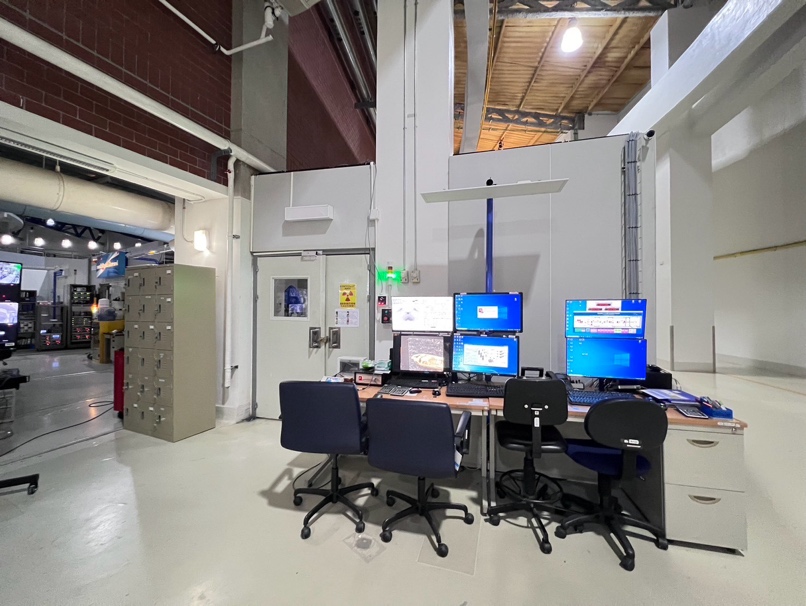 | 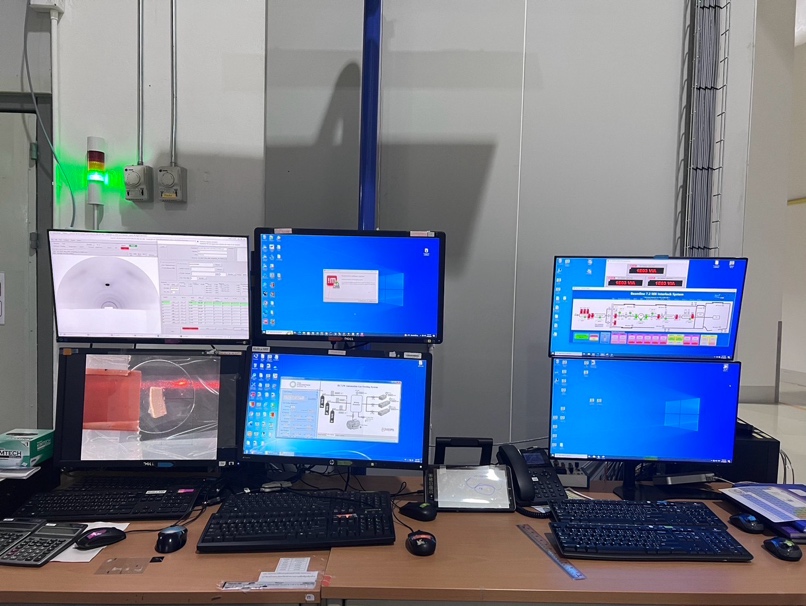 |
| --- | --- |
| 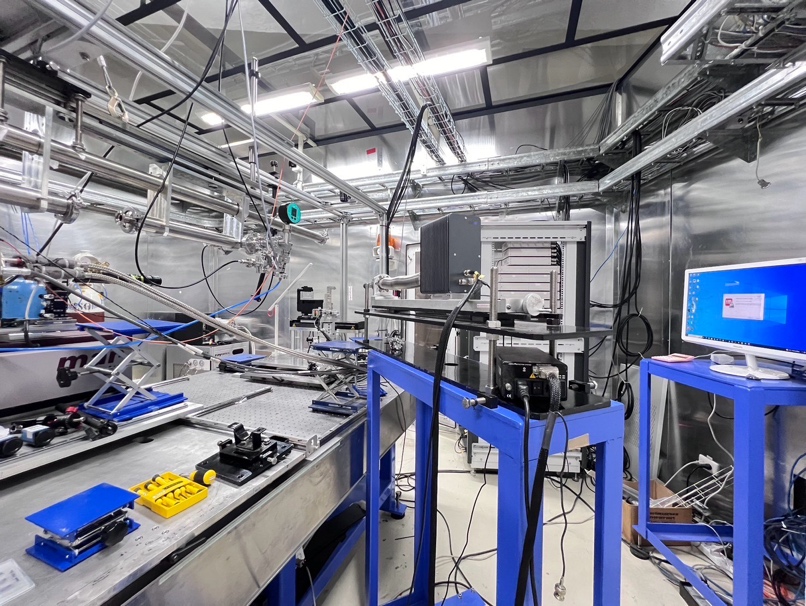 | 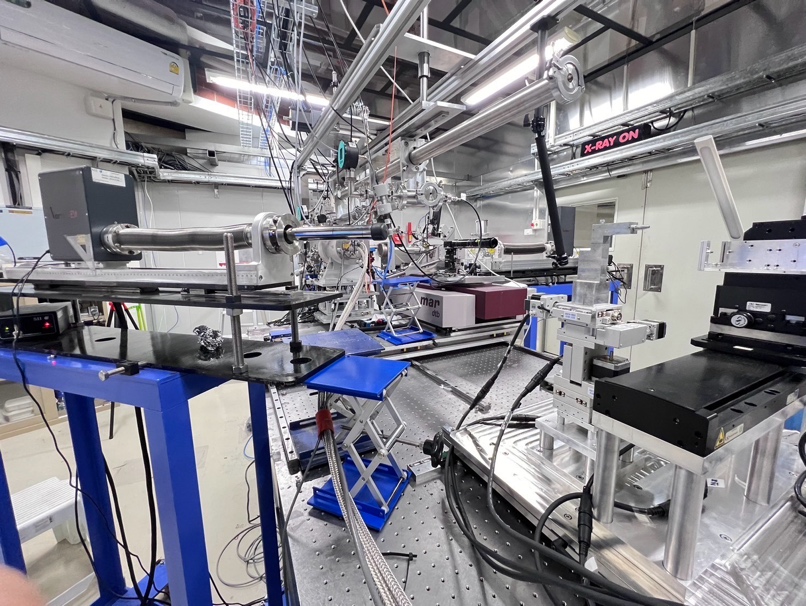 |
| 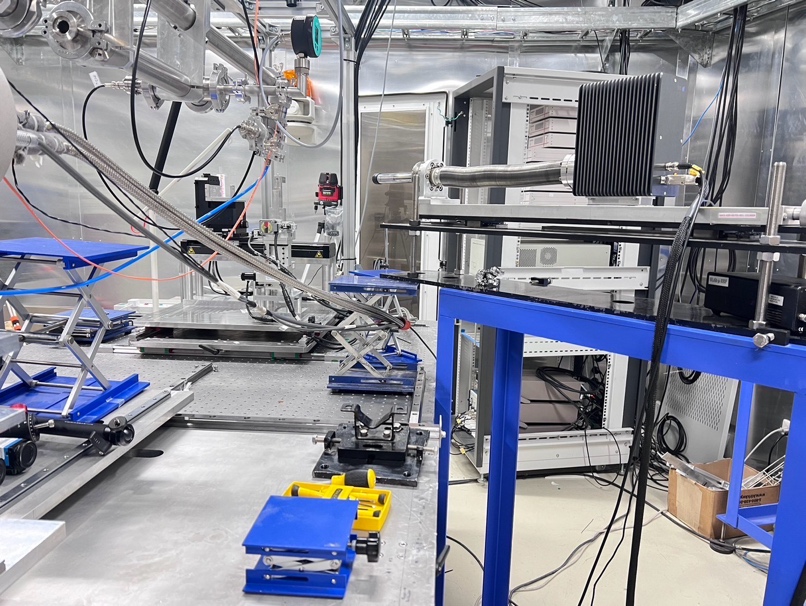 | 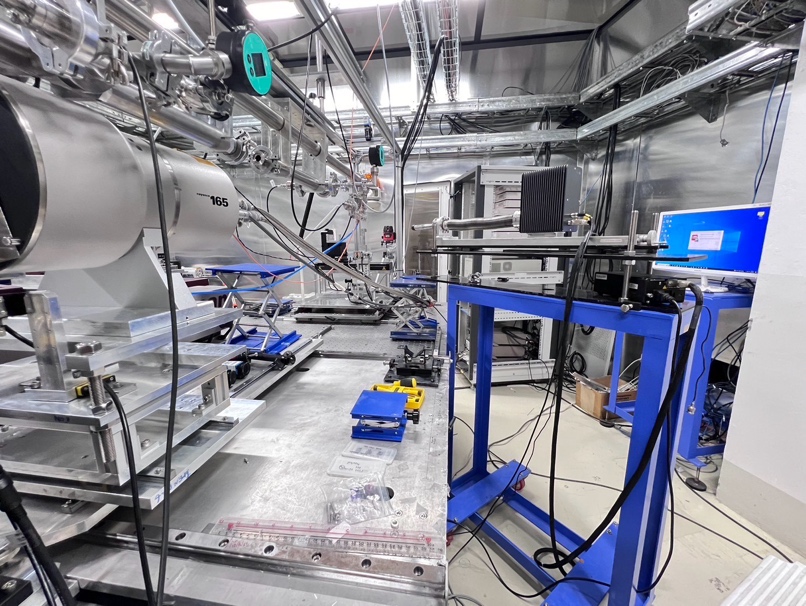 |

Fig. S2. The BL7.2W beamline at SLRI, originally designed for Macromolecular Crystallography, underwent enhancements including the addition of a Si-drift X-ray detector, sample micro-stage, and polycapillary X-ray lens. These upgrades enable the performance of experiments involving XRF imaging and micro-beam X-ray absorption spectroscopy (μXAS) techniques.

Table S1. Infrared absorption bands and vibrational modes of detected OFGs on TSP collected at WAQOS (Pongpiachan et al., 2022).

| Absorbance band (cm^-1^) | Vibration mode | Species |
| --- | --- | --- |
| 3000–2800 | R-H | Aliphatic carbons |
| 1750–1700 | C=O | Carbonyl species - Hemicellulose - Pectin - Lectin |
| 1660–1620 | NO_2_ asymmetric stretching  (R-ONO_2_) | Organo-nitrates |
| 1550–1500 | NO_2_ asymmetric stretching (Arom-NO_2_) | Aromatic nitro compounds |
| 1485–1390 | NH_4_ ^+^ | Ammonium ions |
| 1390 | *ν*-asym (CO_3_^2-^) | Carbonate |
| 1345–1315 | *ν*-asym (NO_3_^-^) | Nitrate ions |
| 1180–900 | S=O stretching  - SO_4_^-^  - HSO_4_^-^ | Sulfate species - Sulfate ions - Bisulfate ions |
| 879 | *ν*-asym (CaCO_3_ ^2-^) | Calcium carbonate |
